# Phosphoproteomics reveals therapeutic targets of esophageal squamous cell carcinoma

**DOI:** 10.1038/s41392-021-00682-5

**Published:** 2021-11-12

**Authors:** Yi Li, Bin Yang, Yanchun Ma, Xiaojun Peng, Zhiyu Wang, Bin Sheng, Zichao Wei, Yongping Cui, Zhihua Liu

**Affiliations:** 1 State Key Lab of Molecular Oncology, National Cancer Center/Cancer Hospital, Chinese Academy of Medical Sciences and Peking Union Medical College, Beijing, 100021 China; 2 Department of Pathology, Key Laboratory of Cellular Physiology (Shanxi Medical University), Ministry of Education, Taiyuan, 030001 Shanxi China; 3 Department of Tumor Surgery, Shanxi Cancer Hospital, Taiyuan, 030013 Shanxi China; 4 Department of Bioinformatics, Jingjie PTM Biolab Co. Ltd, Hangzhou, 310018 China

**Keywords:** Cell biology, Gastrointestinal cancer, Computational biology and bioinformatics

**Dear Editor**,

Esophageal squamous cell carcinoma (ESCC) is one of the most aggressive squamous cell carcinomas and highly prevalent in Asia. The incidence of ESCC is affected by environmental factors (alcohol consumption, tobacco use, etc.) and genetic factors, both result in tumorigenesis by altering proteomic, posttranslational modification (PTM) and metabolic characterization of esophageal epithelial cells. Recently, large-scale investigations of ESCC have been performed, focusing on the discovery of new driver mutations. However, the mechanisms underlying ESCC development remain unclear, and comprehensive protein profiling of ESCC is necessary to understand its underlying mechanisms.

So far, the approaches to the treatment of ESCC patients mainly include endoscopic therapy, surgery and chemoradiotherapy. However, most patients especially with poor prognosis ESCC still lack effective targeted treatment. Phosphorylation is of common occurrence in cells and leads to a cascade of downstream signaling events important for cell and dysregulation of this process has been implicated in cancer. By now, great progress has been made in developing therapeutic drugs that antagonize the activity of kinases that are aberrantly activated in cancer. It stands to reason that the implementation of phosphoproteomics may provide greater clues to develop effective therapeutic targets. Here, we selected 94 surgically resected primary tumor tissues (T) and 24 non-tumor esophageal tissues (N) from 94 cases of intermediate- and advanced-stage (TNM II–IV stage) ESCC and performed an extensive proteomic and phosphoproteomic characterization with consistent quality control using the iTRAQ technique (Supplementary Fig. S1). A total of 9,042 proteins as well as 26,892 phosphosites were identified (Supplementary Table S1). On average, 5,049 proteins and 7,064 phosphosites quantified per non-tumor esophageal tissues were quantified and 4,875 proteins as well as 9,376 phosphosites per tumor esophageal tissues were quantified (Supplementary Fig. S2a). Principle component analysis (PCA) showed the non-tumor tissues were clustered together and clearly separated from the tumors based on the proteomic (Fig. 1a) and phosphoproteomic (Fig. 1b) data. Proteomics data identified a total of 556 differentially expressed proteins (DEPs), including 227 upregulated and 329 downregulated proteins (Supplementary Fig. S2b). The top downregulated and upregulated DEPs were annotated as extracellular and nuclear proteins, respectively (Supplementary Fig. S2c). Phosphoproteomic data identified a total of 1691 differentially expressed phosphorylation sites (DEPSs), including 695 upregulated and 996 downregulated DEPSs in 491 and 447 differentially expressed phosphorylation proteins (DEPPs), respectively (Supplementary Fig. S2d). The upregulated DEPSs were mainly enriched in the nucleoplasm and nucleoli (Supplementary Fig. S2e). ESCC were mainly characterized by elevated proteomic and phosphoproteomic levels in the spliceosome pathway and the cell cycle pathway, and lowered ECM-receptor interaction and focal adhesion pathway (Supplementary Fig. S3).

**Fig. 1:**
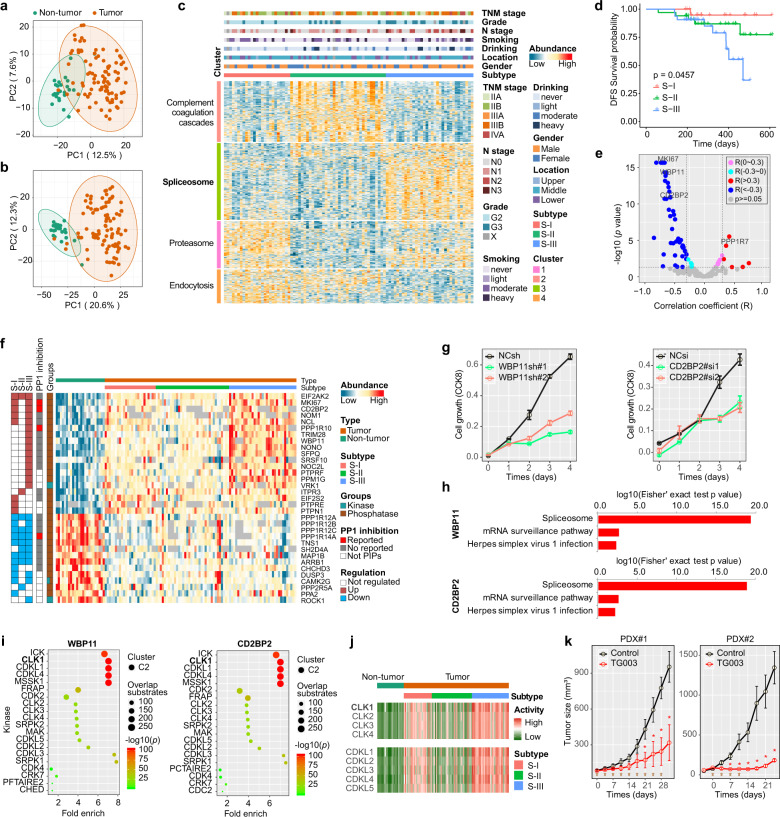
**a**, **b** PCA scatter plots of proteomic (**a**) and phosphoproteomic (**b**) datasets in 94 tumors and 24 non-tumor tissues. **c** Consensus clustering analysis of proteomic profiling identifies 3 proteomic subtypes:S-I (red), S-II (green), and S-III (blue). The associations of proteomic subtypes with clinical characteristics (TNM stage, grade, age, drinking, smoking, etc.) are annotated in the upper panel. Top two KEGG pathways related to these cluster signature proteins are denoted on the left, and annotated according to the KEGG pathway enrichment analysis (detailed in Supplementary Table S3). **d** Kaplan-Meier curves of disease-free survival (DFS) for each proteomic subtype in our cohort. **e** Volcano plot of the correlation coefficient (R) between phosphatase or phosphatase interacting protein (PIP) abundance with inferred phosphatase activity. **f** Differentially expressed phosphatase or phosphatase-PIPs and kinase profiles in three subtypes of ESCC and non-tumor tissues. **g** Effect of WBP11 (left) and CD2BP2 (right) knockdown on ESCC cell proliferation. **h** The top 3 KEGG pathways were enriched by the differentially expressed phosphorylation interactors (DEPIs) of WBP11 (top) and CD2BP2 (bottom). **i** Top 20 kinases in the pairing relationship analysis (PRA) of two PP1 PIPs, WBP11 (left) and CD2BP2 (right) opposed. The shape (dot or square) represents the cluster to which the kinase belonged. The size of dot or square represents the mapping number of proteins, the color of dot or square represents the significance. **j** Differentially inferred activity of CLKs and CDKLs profiles in three subtypes of ESCC and non-tumor tissues. **k** The average volume of tumors (± s.e.m.) of ESCC PDX model #1 and #2 treated with control (PBS) and TG003 at the indicated times. Brown arrows indicated the times of drug treatment

Furthermore, three major proteomic subtypes (S-I, S-II and S-III) in this tumor cohort of ESCC were defined (Fig. 1c and Supplementary Fig. S4). And the S-III had the significantly lowest disease-free survival (DFS) (Fig. 1d) and the most proportion of patients with lymph node metastasis (Supplementary Table S2), and was characterized by elevated proteins and phosphorylation proteins level in spliceosome pathways (Supplementary Fig. S5).

To identify the overactive kinase and explore the druggable targets in ESCC, kinase activity prediction derived from phosphopeptide data. Consistent with high levels of cell cycle activity, nuclear-localized S/T kinase (CDK) (cluster 2) activities in ESCC tumor tissues (especially S-III) were higher than non-tumor tissues (Supplementary Fig. S6a). In contrast to the specificity of kinases due to binding to the specific motifs of their substrates, phosphatases use the following strategies to target their substrates: through their own targeting domains or motifs directly bind to their substrates (such as protein tyrosine phosphatases (PTPs)), and through some phosphatase interacting proteins (PIPs) (also named regulatory subunit) (such as protein phosphatase 1 (PP1), protein phosphatase 2 A (PP2A), etc.).^1^ PP1 is a single-domain hub protein with nearly 200 validated interactors. PIPs can increase the local concentration of PP1 through binding PP1 and control associated PP1 by interference with substrate recruitment or access to the active site. Several PP1 interactome had been performed and identified hundreds of PIPs.^2^ Here, we considered phosphatase and PIPs together as a unit enzyme for predicting its activity by phosphatases or phosphatase-PIPs—substrates enrichment analysis (PhSEA). Except for a small number of PPP1C-PIPs (cluster 4) activity was increased in ESCC, lots of nuclear-localized PPP1C-PIPs (cluster 3) activity was remarkably downregulated in ESCC (especially S-III) (Supplementary Fig. S6b). In addition, our data showed that only a small number of kinase activities were correlated with their abundances, which was correlated more with the phosphorylation level at particular Tyr/Ser/Thr sites (Supplementary Fig. S6c-e), which was which frequently downregulated in ESCC (Supplementary Fig. S6f). Moreover, only a few PIPs (PPP1R7, etc.) positively correlated with PP1-PIP activity, a majority of PIPs were negative correlated with PP1-PIP activity functioned as PP1 inhibitor (Fig. 1e) and some of them, such as CD2BP2, WBP11 and MKI67 etc. have been reported to inhibit PP1 and considered as inhibitors of PP1.^2,3^ Both CD2BP2 and WBP11 had been reported to involve in spliceosome assembly and pre-mRNA splicing^3,4^ and were upregulated in S-III ESCC (Fig. 1f). Knockdown of CD2BP2 and WBP11 remarkably inhibited the KYSE150 and KYSE30 growth (Fig. 1g and Supplementary Fig. S7a-b), which suggested that these two PP1 inhibitors played a key role in proliferation. Moreover, the ESCC patients with high CD2BP2 had the significantly lower DFS (Supplementary Fig. S7c). To further understand their functional role in tumor development, the KEGG pathways of PPP1C-PIPs associated differentially expressed phosphorylation interactors (DEPIs) were generated (Supplementary Fig. S7d-e), and the top pathway enriched by the DEPIs of PPP1C-CD2BP2 and PPP1C-WBP11 both was the spliceosome pathway (Fig. 1h).

The phosphatases with corresponding kinases well coordinate to ensure phosphorylation homeostasis of proteins. Here, the corresponding kinases of each phosphatase were analyzed by paired relationship analysis (PRA). As shown in Fig. 1i, the substrates of PPP1C-CD2BP2 and -WBP11 mainly overlapped with the substrates of CDC-like kinase 1(CLK1), cyclin-dependent kinase-like (CDKL), etc., which suggested that CD2BP2 and WBP11 functioned as enhancer of these CLK1 and CDKL kinases. Consistently, inferred activity of CLK1 was mostly increased in S-III (Fig. 1j), and CLK1 DEPIs were also enriched in the spliceosome pathway (Supplementary Fig. S7f). CLK1 phosphorylate serine- and arginine-rich (SR) proteins (such as SRSF1, SRSF3) of the spliceosomal complex and enable SR proteins to control RNA splicing.^5^ Our data showed that SRSF family proteins and HNRNPD phosphorylation were upregulated in ESCC S-III (Supplementary Fig. S7g-h). Patients of S-III have the worst prognosis after surgery, and should receive further targeted therapy. To evaluate the potential of CLK1 for further ESCC treatment, TG003 (CLK1 inhibitor) was used and injected intraperitoneally into three ESCC patient-derived tumor xenograft (PDX) mouse models. Treatment with moderate TG003 dosage had no effect on mice body weight (Supplementary Fig. S8a-b), and reduced tumor growth in two PDX models, especially in PDX#2 (Fig. 1k). However, moderate dosage of TG003 had no effect on tumor growth in PDX#3, only high dosage of TG003 had an effect on reducing tumor growth (Supplementary Fig. S8c). Moreover, the most TG003-sensitive PDX#2 has the highest levels of WBP11 and CD2BP2 (Supplementary Fig. S8d), and the reason for the TG003-insensitive PDX#3 may be due to its higher proliferation rate, although it has the same level of WBP11 and CD2BP2 as PDX#1. In addition, TG003 had more advantage in treatment of ESCC with CD2BP2 and WBP11 high level than CDK4/6 inhibitor (palbocicib) (Supplementary Fig. S8b).

Overall, our data revealed that S-III ESCC with the worst prognosis after surgery were characterized by elevated proteomic and phosphoproteomic levels in the spliceosome pathway, and some of PP1 inhibitors (CD2BP2, WBP11, etc.) functioning as enhancer of CLK1 kinase were upregulated in S-III and involved in ESCC development; and CLK1 might represent a new promising therapeutic target in attempts to improve the poor prognoses of ESCC.

## Supplementary information

Supplementary Materials

Dataset 1

Dataset 2

Dataset 3

Dataset 4

Dataset 5

Dataset 6

## Data Availability

The raw proteome data have been deposited to the integrated proteome resource iProX (Project accession: IPX0002466000).
