# Supplementary material for: Phosphoproteomics reveals therapeutic targets of esophageal squamous cell carcinoma
Source: Signal Transduct Target Ther. 2021 Nov 12;6:381. doi: 10.1038/s41392-021-00682-5 (PMC8585941; doi:10.1038/s41392-021-00682-5)
Supplement: Supplementary file 1 — Supplementary Materials [file 41392_2021_682_MOESM1_ESM.docx]

Supplementary Materials for

Phosphoproteomics Reveals Therapeutic Targets of Esophageal Squamous Cell Carcinoma

Yi Li, Bin Yang, Yanchun Ma, Xiaojun Peng, Zhiyu Wang, Bin Sheng, Yongping Cui & Zhihua Liu

Correspondence to: Zhihua Liu (liuzh@cicams.ac.cn) or Yongping Cui (cuiyp@sxmu.edu.cn)

**This PDF file includes:**

Materials and Methods

Figure. S1 to S8

Supplementary Table S1 to S6 (attached as single excel files)

**Materials and Methods**

Esophageal Squamous Cell Carcinoma (ESCC) Sample Preparation

All 94 ESCC primary tumor tissues and 24 paired non-tumour esophageal tissues from these 94 cases of intermediate- and advanced-stage (TNM II–IV stage) ESCC were used in this study, with the approval of the Institutional Review Board of the Chinese Academy of Medical Sciences Cancer Hospital and the ethical committees of the Shanxi Medical University. Medical record was obtained for all 94 ESCC patients (surgery from May 2017 to July 2018). The immune cell infiltration of each tumor was examined on haematoxylin and eosin stained slides. The protein sample preparation and liquid chromatography-tandem mass spectrometry (LC-MS/MS) detection using the isobaric tags for relative and absolute quantitation (iTRAQ) technique were performed in Jingjie PTM Biolab Co. Ltd (Hangzhou, China). The fresh samples were shipped to Jingjie PTM Biolab in dry ice and kept frozen at -80 °C freezer until processing of protein extraction. The tissues were grinded by liquid nitrogen into cell powders and then four volumes of lysis buffer (8 M urea, 1% Protease Inhibitor Cocktail) was added to the cell powder, followed by sonication three times on ice using a high intensity ultrasonic processor. After centrifugation at 12,000 g at 4 °C for 10 min, the supernatant was collected. For following digestion, the protein solution was reduced with 5 mM dithiothreitol for 30 min at 56 °C and alkylated with 11 mM iodoacetamide for 15 min at room temperature in darkness. The protein sample was then diluted by adding 100 mM triethylammonium bicarbonate (TEAB) to urea concentration less than 2M. Finally, trypsin was added at 1:50 trypsin to protein mass ratio for the first digestion overnight and 1:100 trypsin to protein mass ratio for a second 4 hours digestion.

iTRAQ Labeling (and Affinity Enrichment for Phosphorylation)

After trypsin digestion, peptides were desalted by Strata X C18 SPE column (Phenomenex, Torrance, CA) and vacuum-dried. Peptides were redissolved in 0.5 M TEAB and processed according to the protocol for iTRAQ kit. Briefly, one unit of iTRAQ reagent was thawed and reconstituted in acetonitrile. The peptide mixtures were then incubated for 2 hours at room temperature and pooled, desalted and dried by vacuum centrifugation.

To enrich phosphorylated peptides, the peptide mixtures were first incubated with immobilized metal affinity chromatography (IMAC)-TiQ_2_ (for phosphoserine and phosphothreonine) microspheres suspension with vibration in loading buffer (50% acetonitrile/6% trifluoroacetic acid) The IMAC microspheres with enriched phosphopeptides were collected by centrifugation, and the supernatant was removed. To remove nonspecifically adsorbed peptides, the IMAC microspheres were washed with 50% acetonitrile/6% trifluoroacetic acid and 30% acetonitrile/0.1% trifluoroacetic acid, sequentially. To elute the enriched phosphopeptides from the IMAC microspheres, elution buffer containing 10% NH_4_OH was added and the enriched phosphopeptides were eluted with vibration. The supernatant containing phosphopeptides was collected and lyophilized for LC-MS/MS analysis. For enriching phosphotyrosine peptides, tryptic peptides dissolved in NETN buffer (100 mM NaCl, 1 mM EDTA, 50 mM Tris-HCl, 0.5% NP-40, pH 8.0) were incubated with pre-washed anti-phosphotyrosine antibody beads (PTM-701, PTM Bio) at 4°C overnight with gentle shaking. Then the beads were washed four times with NETN buffer and twice with H_2_O. The bound peptides were eluted from the beads with 0.1% trifluoroacetic acid. Finally, the eluted fractions were combined and vacuum-dried. For LC-MS/MS analysis, the resulting peptides were desalted with C18 ZipTips (Millipore, Billerica, MA) according to the manufacturer’s instructions.

High Performance Liquid Chromatography Fractionation and LC-MS/MS Analysis

The peptides were fractionated into fractions by high pH reverse-phase HPLC using Agilent 300Extend C18 column (5 μm particles, 4.6 mm ID, 250 mm length). Briefly, peptides were first separated with a gradient of 8% to 32% acetonitrile (pH 9.0) over 60 min into 60 fractions. Then, the peptides were combined into 18 fractions and dried by vacuum centrifuging. The peptides were dissolved in 0.1% formic acid (solvent A), directly loaded onto a home-made reversed-phase analytical column (15-cm length, 75 μm i.d.). The gradient was comprised of an increase from 6% to 23% solvent B (0.1% formic acid in 98% acetonitrile) over 26 min, 23% to 35% in 8 min and climbing to 80% in 3 min then holding at 80% for the last 3 min, all at a constant flow rate of 400 nL/min on an EASY-nLC 1000 ultra-high pressure liquid chromatography (UPLC) system.

The peptides were subjected to NSI source, and then followed by tandem mass spectrometry in Q Exactive^TM^ Plus (Thermo Fisher Scientific, San Jose, CA) coupled online to the UPLC. The electrospray voltage applied was 2.0 kV. The m/z scan range was 350 to 1800 for full scan, and intact peptides were detected in the Orbitrap at a resolution of 70,000. Peptides were selected for tandem mass spectrometry using normalized collision energy (NCE) setting as 28%, and then the fragments were detected in the Orbitrap at a resolution of 17,500. A data-dependent procedure that alternated between one mass spectrometry scan followed by 20 tandem mass spectrometry scans with 15.0s dynamic exclusion. Automatic gain control (AGC) was set at 5E4. Fixed first mass was set as 100 m/z.

Lentivirus-Mediated Knockdown of WBP11 in ESCC Cells

The pSIH1 lentiviral shRNA expression vector was used to construct shRNA expressing plasmids. The lentiviruses encoding shRNA sequences for WBP11 (sh#1: GCCACAGATCACTAATCCCAA; sh#2: GCCACCCTCTATCCTAAAGAA) and control shRNA were produced according to the manufacturer’s instructions. KYSE30 and KYSE150 cells were infected with these lentiviruses, and the stable knockdown cells were obtained for the following 1 μg/ml puromycin selection for one week.

Proliferation Assay

To confirm that PPP1C-regulators confer the growth advantage for ESCC cells, the KYSE30 & KYSE150 cells with stable knockdown WBP11 were established by shRNA lentiviral plasmid construct infection. In addition, the KYSE30 & KYSE150 cells with transient knockdown were transfected with CD2BP2 Stealth siRNA (Invitrogen, Carlsbad, CA, Catalog # 1299001). Then, the knockdown cells were seeded into 96-well plates for 2 days. CCK8 at a final concentration of 10% was added to each well. The absorbance was measured at 450 nm. The data was visualized using R software.

Patient-Derived Xenograft (PDX) Mouse Models

All protocols for mouse experiments were approved by the Chinese Academy of Medical Sciences Cancer Hospital Animal Care and Use Committee. The pieces of ESCC tumor tissues were transplanted into the right flanks of male severe-combined immunodeficiency NOD-SCID IL-2 receptor gamma null (NSG) (NOD^prkdc-/-,IL-2Rg-/-^) mice (5~6 weeks old) (GemPharmatech Co.Ltd, China) subcutaneously. Once the subcutaneous tumor reached 1 cm in diameter, it was cut into pieces of 2×2×2mm^3^ and then subcutaneously implanted into the flanks of 5~6 weeks old NSG mice. When the tumor size reaching about 70~80 mm^3^, the mice were randomly divided into the 2 groups and treated intraperitoneally with following drug in PDX #1 and #2: (1) control (PBS), (2) TG003 (0.6mg/kg). In addition, the mice in PDX#3 were randomly divided into the 3 groups: (1) control (PBS), (2) TG003 (0.6mg/kg) and (3) TG003 (1.2mg/kg). After experiment finished, the mice were sacrificed. Tumor size was measured using a caliper, and calculated using the formula: tumor volume = 0.5 × length × (width)^2^. The mean of tumor sizes was visualized using R software.

Annotation Methods

Gene Ontology (GO) annotation of the proteome was derived from the UniProt-GOA database (http://www.ebi.ac.uk/GOA/), and Kyoto Encyclopedia of Genes and Genomes (KEGG) database was used to annotate proteins pathway. In addition, Wolf PSORT (a subcellular localization predication software) was used to predict subcellular localization. For providing the more accurate and higher-resolution insights into the spatiotemporal distribution, the proteins were annotated with the localization data of The Human Protein Atlas (http://www.proteinatlas.org/) derived from antibody-based profiling by immunofluorescence confocal microscopy, in combination with secreted proteins annotated by Wolf PSORT because extracellular proteins cannot been annotated effectively by The Human Protein Atlas database.

Protein Alterations and Hyper/Hypo-Phosphorylated Proteins in ESCC

A total of 9,402 proteins (<1% FDR) as well as 26,892 phosphopeptides (Y: 2,791; S/T: 24,101) (<1% FDR and modified peptide score > 40 and site localization probability > 0.75) were identified with 4,911 proteins and 8,906 phosphopeptides quantified per tumor and non-tumor esophageal tissues (**Supplementary Table S1**). Proteins and phosphosites (5,054 proteins and 8,541 phosphosites) observed at least in 50 samples were used for DEPs analysis between tumor and non-tumor tissues. Student’s T-test was used to calculate significant p value. The 1637 differentially expressed proteins (DEPs) between tumor (T) and non-tumor (N) esophagus tissues with statistical significance (p < 0.05 and fold change (T/N) >1.5 or <1/1.5) were identified, including 227 upregulated and 329 downregulated proteins. In addition, a total of 1691 differentially expressed phosphorylation sites (DEPSs) between T and N tissues with statistical significance (*p* < 0.05 and T/N >1.5 or <1/1.5) were identified, including 695 upregulated and 996 downregulated DEPSs in 491 and 447 proteins (DEPPs) respectively. The DEPs and DEPPs identified in the comparison between the tumor and non-tumor samples were then subjected to functional enrichment analysis, and the statistical significance of the functional enrichment was determined by Fisher’s exact test.

Identification of Proteomic Subtype in ESCC and Signature Proteins and Phosphorylation Sites for Subtypes

The protein expression matrix of the 94 tumor samples was used to identify the proteomic subtypes using the 𝑘-means consensus clustering method. Before the consensus clustering analysis, we filtered out 2405 proteins with quantified in all 94 samples. Then, we performed a centered log ratio transformation to facilitate the interpretation of the expression data. The resulting dataset was transformed into 1,000 bootstrap sample data sets with a probability of 0.8 for selecting any sample. The bootstrap data sets were clustered using 𝑘-means clustering with up to 6 clusters. The consensus matrix for 𝑘=2,3,4,5,6 clusters were shown in **Supplementary Fig. S4**. The proportion of ambiguous clustering (PAC) scores ^2^ were measured for consensus clustering runs. Based on PAC analysis result, proteome clusters were defined using 𝑘-means consensus clustering results with 𝑘=3.

To identify the molecular signature of proteomic subtypes, the expression data was performed a centered log ratio transformation and filtered out the proteins quantified in at least 50 samples to identify signature proteins and phosphorylation sites for subtypes. For each subtype, DEPs (|log2 ratio| > 1, FDR<0.01) between tumor and non-tumor were considered as signature proteins or phosphorpeptide of subtype. Detail data shown in **Supplementary Table S1**.

Correlations between Proteomic Subtypes and Clinical Features

The association between the proteomic subtypes and clinical information was examined by fisher’s exact test. Kaplan–Meier survival curve was used to compare the disease-free survival (DFS) among the proteomic subtypes. S-I patients had the highest DFS and the fewest proportion of patients with lymph node metastasis, advanced-stage, poor differentiation, we used S-I tumors as control variable and then compared with S-II and S-III tumors to evaluate the poor prognostic power of these tumors by Pearson's chi-squared test and Fisher's exact test, and the results were showed in **Supplementary Table S2**. All statistical analyses were performed in R.

Kinase Annotation and Activity Prediction

We predicted ESCC-associated kinase activity based on the following method. Here, we used iGPS (GPS algorithm with the interaction filter, or *in vivo* GPS) mainly for the prediction of *in vivo* ssKSRs (site-specific kinase-substrate relations).^3^ The upstream kinase annotation were showed in **Supplementary Table S1**. We then annotated log2 ratio based-on ranked phosphopeptide lists with predicted upstream kinases from the database of kinase substrate interactions. The kinase activity was defined as these sites in each of tumor and normal tissue by NES (normalized enrich score) calculated by gene set enrichment analysis (GSEA) method (**Supplementary Table S4**).

Phosphatase Annotation and Activity Prediction

Phosphatases mainly use three strategies to physically target their substrates: (i) through its targeting domains and Small Linear Motifs (SLiM) directly binding to its substrates, (ii) regulatory subunits or scaffold proteins binding with phosphatases.^4^ Thus, the holoenzyme consisting of phosphatase and numbers of regulatory subunits was a complete functional unit. Therefore, we considered phosphatase and regulators together as a unit for predicting phosphatase activity. There are three types of phosphatases in our predicted phosphatase activity model. Type 1: Catalytic and targeting domain/motif in same protein, including protein tyrosine phosphatases (PTPs), protein phosphatase 2B (PP2B), protein phosphatase 5 (PP5); Type 2: Catalytic and targeting domain/motif in different proteins binding together, including protein phosphatase 1 (PP1). These phosphatases were named by phosphatase catalytic domain combined with its regulatory subunits or PP1 interaction proteins. (such as PPP1C-PPP1R10); Type 3: Catalytic and targeting domain/motif in different proteins forming complex through scaffold protein, including protein phosphatase 2A (PP2A), protein phosphatase 4 (PP4), protein phosphatase 6 (PP6). These phosphatases were also named by phosphatase catalytic domain combined with its regulatory subunits (such as PPP2C-PPP2R2A, PPP4C-PPP4R1, PPP6C-PPP6R1, etc.). For each phosphoprotein identified by MS was searched for predicted upstream phosphatases/or phosphatase-PIPs depending on whether an interaction exists between them. All the interactions between them with phosphatase and regulator or scaffold proteins were derived from Search Tool for the Retrieval of Interacting Genes/Proteins (STRING) Databases (https://string-db.org/) (v10.0.) ^5^ with high confidence (interaction score > 0.70). The upstream phosphatases/or phosphatase-PIPs annotation were showed in **Supplementary Table S1**. The highest / lowest of value of phosphorylation site among the all phosphorylation sites in same protein was used to rank the upregulated/downregulated phosphorylation protein. Then we calculate the score of phosphatase/or phosphatase-regulator activity based on gene set enrichment analysis (GSEA) method (**Supplementary Table S5**). Here we named this method for calculating the activity of phosphatases as Phosphatases/or Phosphatase-PIPs—Substrates Enrichment Analysis (**PhSEA**).

Analysis of Correlation between Activity and Abundance of kinase or phosphatease

The association between kinase and phosphatase activity with kinase and phosphatase/ phosphatase-PIP protein abundance was examined with Pearson's correlation coefficients. Total 152 phosphatases, phosphatase-PIPs and 60 kinases were involved in this correlation analysis. Before the correlation analysis, log-transformed abundance of identified kinase/phosphatase or phosphatase-PIPs in 118 proteome samples. Here, we used volcano plot to visualize the activity and protein abundance correlation coefficient and -log10(*p* value).

The Construction of the Protein-Protein Interaction (PPI) Network Associated with ESCC

We utilized the STRING database (v10.0) (https://string-db.org/) ^5^ for constructing the physical interaction network associated with ESCC, and used Cytoscape 3.7.2 to visualize the PPIs network. The proteins were grouped by community membership. All the interactions between the DEPPs with statistical significance (*p* < 0.05 and fold change (T/N) >1.5 or <1/1.5) with phosphatase and regulatory or scaffold proteins were derived from STRING databases with high confidence (interaction score > 0.70). Maximum fold change of phosphorylation site was used to represent overall phosphorylation abundance change of proteins. Phosphorylated proteins directly interacting with phosphatase and regulatory or scaffold proteins were extracted. Those protein enriched KEGG pathways were filterd out by Fisher’s exact test method.

The Paired Relationship Analysis (PRA) between Kinases and Phosphatases

Protein phosphorylation level is maintained by both kinase and phosphatase. There were co-regulated substrates between specific kinases and phosphatases or phosphatase-PIPs. To discover the pairing relationship between kinases and phosphatases or phosphatase-PIPs through the numbers of their co-regulating substrates, Fisher’s exact test method was used to assess the reliability of the paired relationship. While *p* < 0.05, pairing relationship was considered significance. The PRA results of kinases and PPP1C-WBP11 and PPP1C–CD2BP2 were showed in **Supplementary Table S6**.

| **Phosphosites** | | **Phosphatase substrates** | |
| --- | --- | --- | --- |
|  |  | **yes** | **no** |
| **Kinase substrates** | **yes** | *a* | *b* |
|  | **no** | *c* | *d* |

Fisher showed that the probability of obtaining any such set of values was given by the hypergeometric distribution:

$$p=\left( \frac{a+b}{a} \right)(\frac{c+d}{c})/(\frac{n}{a+c})$$

**REFERENCES:**

1 Cui Y, Chen H, Xi R *et al.* Whole-genome sequencing of 508 patients identifies key molecular features associated with poor prognosis in esophageal squamous cell carcinoma. *Cell Res* 2020.

2 Senbabaoglu Y, Michailidis G, Li JZ. Critical limitations of consensus clustering in class discovery. *Sci Rep* 2014; **4**:6207.

3 Song C, Ye M, Liu Z *et al.* Systematic analysis of protein phosphorylation networks from phosphoproteomic data. *Mol Cell Proteomics* 2012; **11**:1070-1083.

4 Sacco F, Perfetto L, Castagnoli L, Cesareni G. The human phosphatase interactome: An intricate family portrait. *FEBS Lett* 2012; **586**:2732-2739.

5 Szklarczyk D, Gable AL, Lyon D *et al.* STRING v11: protein-protein association networks with increased coverage, supporting functional discovery in genome-wide experimental datasets. *Nucleic Acids Res* 2019; **47**:D607-D613.

**Supplementary Fig. S1** Quality assessment of proteomic and phosphoproteomic data. (**a-b)** Distribution of log2-transformed the abundance of identified proteins in 108 proteome (**a**) and phosphoproteome (**b**) samples. Green box presents non-tumour samples (n = 24), red box presents tumour samples (n = 94). The middle bar represents the median, and the box represents the interquartile range; bars extend to 1.5× the interquartile range. (**c-d)** Pearson correlations of non-tumour and tumour samples in proteome (**c**) and phosphoproteome (**d**) data are shown.

**Supplementary Fig. S2.** The proteomic and phosphoproteomic characteristics of ESCC.

**(a)** On average, 4,875 proteins per tumor and 5,049 proteins per non-tumor esophageal tissues were quantified, as well as 9,376 phosphosites per tumor and 7,064 phosphosites per non-tumor esophageal tissues were quantified. (**b)** The 558 differentially expressed proteins (DEPs) (227 upregulated and 329 downregulated proteins) were identified in ESCC. **(c)** Subcellular distribution of downregulated and upregulated DEPs in ESCC. (**d)** The 1691 DEPSs with statistical significance, including 695 upregulated and 996 downregulated DEPSs in 491 and 447 proteins (DEPPs) in ESCC, respectively. (**e**) Enrichment analysis of subcellular distribution of differentially expressed phosphorylation sites (DEPSs) annotated with the localization data of The Human Protein Atlas in combination with secreted proteins annotated by Wolf PSORT. The size of dot represents the mapping number of protein, the colour of dot represents the significance.

**Supplementary Fig. S3** The diagram summarized relevant ESCC-associated signatures and signalling pathways involved in tumor development and progression.

**Supplementary Fig. S4** The optimal clustering of proteome, as determined using Pearson correlation as distance metric. K (k=2 to 6), number of clusters. Consensus values range from 0 (never clustered together) to 1 (always clustered together) marked by white to dark blue and consensus cumulative distribution function (CDF) plot shows the cumulative distribution functions of the consensus matrix for each k (indicated by colors). The “proportion of ambiguous clustering” (PAC) curve showed that the optimal clusters is 3 (k=3).

**Supplementary Fig. S5** KEGG pathway and subcellular localization enrichment analysis of ESCC subtype-associated proteins and phosphorylation proteins.

(**a**) Enriched KEGG pathways of ESCC subtype-associated protein and phosphorylation proteins that are significantly altered in tumors as compared with non-tumor tissues. The size of dot represents the mapping number of proteins, the colour of dot represents the significance. (**b**) Subcellular localization enrichment of enriched ESCC subtype-associated proteins and phosphorylation proteins that are significantly altered in tumors as compared with non-tumor tissues. The size of dot represents the mapping number of proteins, the colour of dot represents the significance.

**Supplementary Fig. S6** The regulation of kinase and phosphatase in ESCC.

(**a-b**) Heatmap of kinase (**a**) and phosphatase or phosphatase-regulatory (**b**) activity in ESCC. The major kinase or phosphatase groups of these signature proteins are denoted on the right and the proportion of kinases or phosphatases of this major group in all these signature proteins were given in parentheses. (**c**) Volcano plot of the correlation coefficient (R) between kinase protein abundance with its inferred activity. (**d**) Volcano plot of the correlation coefficient (R) between kinase phosphorylation site abundances with its inferred activity. (**e**) The correlation coefficient with 95% confidence interval of ERBB family protein and phosphorylation with its activity. (**f**) Receptor tyrosine kinases (RTKs) phosphorylation profiles in ESCC.

**Supplementary Fig. S7** (**a**) Effect of WBP11 knockdown on KYSE30 cell proliferation. (**b**) Effect of CD2BP2 knockdown on KYSE30 cell proliferation. (**c**) Kaplan-Meier curves for disease-free survival (DFS) in ESCC patients with low and high expression of CD2BP2. (**d**) Network representation of WBP11 and its differentially expressed phosphorylation interactors (DEPIs) (left) and mapped KEGG pathways (right). (**e**) Network representation of CD2BP2 and its differentially expressed phosphorylation interactors (DEPIs) (left) and mapped KEGG pathways (right). The colour of dot represents fold change of DEPIs in ESCC. Red represents upregulation in ESCC and blue represents downregulation in ESCC. (**f**) Networks representation of CLK1 and their differentially expressed phosphorylation interactors (DEPIs) in tumors (left) and mapped KEGG pathways (right). The colour of dot represents fold change of the DEPIs in ESCC. Red represents upregulation in ESCC and blue represents downregulation in ESCC. (**g**) The phosphosite abundances of SRSF family proteins and HNRNPD in ESCC. (h) The CLK1substrate HNRNPD phosphorylation at Ser83 was detected by western blot.

**Supplementary Fig. S8** (**a-c**) The average mouse body weight (left) and volume of tumors (± s.e.m.) (right) of ESCC PDX model #1 (**a**), model #2 (**b**) and model #3 (**c**) treated with control (PBS) and the indicated drugs at the indicated times. Brown arrows indicated the times of drug treatment. The time of mouse with the first drug treatment was condered as zero days. (**d**). The mRNA levels of WBP11 and CD2BP2 in three PDX models were measured by realtime-PCR.
